# Supplementary material for: From sequence to enzyme mechanism using multi-label machine learning
Source: BMC Bioinformatics. 2014 May 19;15:150. doi: 10.1186/1471-2105-15-150 (PMC4229970; doi:10.1186/1471-2105-15-150)
Supplement: Additional file 2 — Java code of ml2db. Additional file ml2db_code.tar.gz contains the Java source code to run the multi-label machine learning experiments and save the results to database. The code’s Javadoc is included. [file 1471-2105-15-150-S2.zip › additional file 2/ml2db/utils/doc/index-files/index-9.html]

I-Index


JavaScript is disabled on your browser.


- Overview
- Package
- Class
- Use
- Tree
- Deprecated
- Index
- Help

- Prev Letter
- Next Letter

- Frames
- No Frames

- All Classes

A B C D E F G H I J K L M N O P Q R S T U V W X Y 


## I

ID - Static variable in class uk.ac.standrews.utils.main.database.DbUtils


implementsInterface(Object, Class) - Static method in class uk.ac.standrews.utils.main.Utils


Initialisable - Interface in uk.ac.standrews.utils.main
:   A class having a boolean variable telling whether the initialisation has
    completed correctly

initialisationIsCorrect() - Method in interface uk.ac.standrews.utils.main.Initialisable


initialisationIsCorrect() - Method in class uk.ac.standrews.utils.main.Initialised


initialise(String, DbManager) - Method in class uk.ac.standrews.utils.main.database.Table


initialise(String, DbManager, Vector<TableColumn>) - Method in class uk.ac.standrews.utils.main.database.Table


Initialised - Class in uk.ac.standrews.utils.main


Initialised() - Constructor for class uk.ac.standrews.utils.main.Initialised


insert(DbConn, String) - Static method in class uk.ac.standrews.utils.main.database.DbUtils
:   Executes sql insert and returns whether the insert was successful.

insertReturnsKey(TableRow) - Method in class uk.ac.standrews.utils.main.database.TableWriter
:   Executes an sql 'insert into' operation.

intArrayToString(int[]) - Static method in class uk.ac.standrews.utils.main.stringutils.StringUtils


INTEGER - Static variable in class uk.ac.standrews.utils.main.database.SqlUtils
:   Basic data type: integer (passes without error a parseInteger action)

INTEGER\_SQL\_DATATYPE - Static variable in class uk.ac.standrews.utils.main.database.DbUtils


interactiveError(boolean, String) - Static method in class uk.ac.standrews.utils.main.guiutils.GuiUtils
:   Emits a popup if the mode is interactive or writes to System.err if non
    interactive

interactiveMessage(boolean, String) - Static method in class uk.ac.standrews.utils.main.guiutils.GuiUtils
:   Emits a popup if the mode is interactive or writes to System.out if non
    interactive

internalKeys() - Static method in class uk.ac.standrews.utils.test.Data


internalNumericalValues() - Static method in class uk.ac.standrews.utils.test.Data


internalOnesAndEmptyStringValues() - Static method in class uk.ac.standrews.utils.test.Data


internalValues() - Static method in class uk.ac.standrews.utils.test.Data


IntFunction - Interface in cern.colt.function
:   Interface that represents a function object: a function that takes a single
    argument and returns a single value.

invertKeysAndValues(OneToManyMap<String, String>) - Static method in class uk.ac.standrews.utils.main.maputils.OneToManyMap


isCorrect() - Method in class uk.ac.standrews.utils.main.database.TableRow


isDash(String) - Static method in class uk.ac.standrews.utils.main.stringutils.StringUtils
:   check if the string is a dash

isEmpty(ResultSet) - Method in class uk.ac.standrews.utils.main.database.TableReader
:   Returns true if the resultset is null or empty.

isEmpty() - Method in class uk.ac.standrews.utils.main.maputils.OneToManyMap


isMultipleOf(int, int) - Static method in class uk.ac.standrews.utils.main.NumberUtils
:   checks if a number is a multiple of another number.

isNullOrEmpty(String) - Static method in class uk.ac.standrews.utils.main.collectionutils.ListUtils
:   tells if a string is null or empty

isNullOrEmpty(Vector<String>) - Static method in class uk.ac.standrews.utils.main.collectionutils.ListUtils
:   tells if a list is null or empty

isPrimaryKey() - Method in class uk.ac.standrews.utils.main.database.TableColumn
:   Gets the column primary key status

isSubsetOfSuperset(TreeSet<String>, TreeSet<String>) - Static method in class uk.ac.standrews.utils.main.collectionutils.setutils.SetUtils


isSubsetOfSuperset(TreeSet<String>, TreeSet<String>) - Static method in class uk.ac.standrews.utils.main.setutils.SetUtils


iterator1() - Static method in class uk.ac.standrews.utils.test.RegExpUtilsTest


iterator2() - Static method in class uk.ac.standrews.utils.test.RegExpUtilsTest

A B C D E F G H I J K L M N O P Q R S T U V W X Y

- Overview
- Package
- Class
- Use
- Tree
- Deprecated
- Index
- Help

- Prev Letter
- Next Letter

- Frames
- No Frames

- All Classes
